# Integrating *De Novo* Transcriptome Assembly and Cloning to Obtain Chicken Ovocleidin-17 Full-Length cDNA

**DOI:** 10.1371/journal.pone.0093452

**Published:** 2014-03-27

**Authors:** Quan Zhang, Long Liu, Feng Zhu, ZhongHua Ning, Maxwell T. Hincke, Ning Yang, ZhuoCheng Hou

**Affiliations:** 1 National Engineering Laboratory for Animal Breeding and MOA Key Laboratory of Animal Genetics and Breeding, Department of Animal Genetics and Breeding, China Agricultural University, Beijing, China; 2 Institute of Animal Genetics and Breeding, College of Animal Science and Technology, Sichuan Agricultural University, Ya'an, China; 3 Department of Cellular and Molecular Medicine, University of Ottawa, Ottawa, Ontario, Canada; Wageningen UR Livestock Research, Netherlands

## Abstract

Efficiently obtaining full-length cDNA for a target gene is the key step for functional studies and probing genetic variations. However, almost all sequenced domestic animal genomes are not ‘finished’. Many functionally important genes are located in these gapped regions. It can be difficult to obtain full-length cDNA for which only partial amino acid/EST sequences exist. In this study we report a general pipeline to obtain full-length cDNA, and illustrate this approach for one important gene (Ovocleidin-17, OC-17) that is associated with chicken eggshell biomineralization. Chicken OC-17 is one of the best candidates to control and regulate the deposition of calcium carbonate in the calcified eggshell layer. OC-17 protein has been purified, sequenced, and has had its three-dimensional structure solved. However, researchers still cannot conduct OC-17 mRNA related studies because the mRNA sequence is unknown and the gene is absent from the current chicken genome. We used RNA-Seq to obtain the entire transcriptome of the adult hen uterus, and then conducted *de novo* transcriptome assembling with bioinformatics analysis to obtain candidate OC-17 transcripts. Based on this sequence, we used RACE and PCR cloning methods to successfully obtain the full-length OC-17 cDNA. Temporal and spatial OC-17 mRNA expression analyses were also performed to demonstrate that OC-17 is predominantly expressed in the adult hen uterus during the laying cycle and barely at immature developmental stages. Differential uterine expression of OC-17 was observed in hens laying eggs with weak versus strong eggshell, confirming its important role in the regulation of eggshell mineralization and providing a new tool for genetic selection for eggshell quality parameters. This study is the first one to report the full-length OC-17 cDNA sequence, and builds a foundation for OC-17 mRNA related studies. We provide a general method for biologists experiencing difficulty in obtaining candidate gene full-length cDNA sequences.

## Introduction

Messenger RNA sequence information is necessary for a variety of studies including mRNA expression, designing mRNA microarrays and genome annotation. Many genome projects provided new solutions for identifying mRNA sequences after the release of the human genome. However, only 32% of the genomes in the GOLD database are ‘complete’ or ‘closed’, meaning that they contain no gaps [1]. An even smaller number have been “finished” by manually correcting errors and adding annotations. Repetitive elements, sequencing biases and other complicating factors all come together to make some regions difficult or impossible to assemble [2]. It is estimated that bases in the gapped regions can account for about 1.3% (chicken) to 7.2% (rhesus macaque) to 12.8% (Purple Sea Urchin) of the genome for 12 representative high quality draft assemblies of highly studied species [2]. Almost all the major domestic animal reference genomes contained many gapped regions. Obviously, some functionally important genes are located in these gapped/mis-assembled regions.

How do we efficiently obtain those full-length cDNAs for genes which are not yet covered by the reference genome? RNA-Seq provides a new efficient method to obtain the entire transcribed mRNA in such examples. The *de novo* transcriptome assembly method has been extensively used in non-model organisms to obtain transcript sequences [3], [4]. In contrast, researchers generally apply mapping-based methods to detect mRNA structure for model organisms. However, this method will miss important genes that are located in gapped regions of the reference genome. The chicken genome was the first well sequenced domestic animal genome [5] and also contains the least gapped regions [2]. One important gene, *Ovocleidin-17* (*OC-17*), is of intense interest with respect to the chicken eggshell biomineralization process. In this study we use OC-17 as example to illustrate a general framework for cloning and obtaining full-length cDNA sequences to pave the way for OC-17 genetic variation and functional studies.

Biomineralization is an important process in a wide range of scientific disciplines including chemistry, biology, medicine, and materials science [6]. Organic macromolecules are important in the regulation of mineral growth, particularly crystal morphology and particle aggregation. Discovering the mechanisms that regulate biomineral formation will provide important insights for advances in human health, material sciences and biology [7]. All birds and most reptiles lay calcareous eggs, while the avian egg is considered to represent the most advanced amniotic egg in oviparous vertebrates [8]. As a typical biogenic mineral, the avian eggshell has attracted considerable attention due to its very rapid rate of formation, well defined ultrastructure and superior mechanical properties that are important features of eggshell quality [8]. The eggshell is a very good model for understanding the mechanisms underlying the phenomena of CaCO_3_ biomineralization [9]. OC-17 is a major eggshell-matrix specific protein and was the first eggshell-specific matrix protein to be isolated and characterized [10]. Chicken OC-17 is one of the best candidates to control and regulate the deposition of calcium carbonate in the calcified eggshell layer [9]. For these reasons, the crystal structure of OC-17 protein has been determined [9]; computer simulations based on this structure suggest that OC-17 protein may act as a catalyst in the transformation of amorphous calcium carbonate to calcite crystals [11]. However, in spite of significant efforts, it has not yet been cloned, and nothing is known about the regulation of OC-17 mRNA expression [8]. Only 20 amino acids of OC-17 are found on the ChrUn in the chicken reference genome. This is a relatively frequent case in biology where researchers know the amino acid sequence, have conducted many protein-based studies, but lack mRNA sequences for expression studies.

In this study we illustrate a novel approach to this problem by combining *de novo* transcriptome assembly and RACE, using the successful example of obtaining the OC-17 full-length cDNA. In doing so, we provide a general method for biologists experiencing difficulty in obtaining full-length cDNAs.

## Materials and Methods

### Ethics Statement

Animal experiments were approved by the Animal Care and Use Committee of China Agricultural University. Euthanasia was performed by cervical dislocation in order to quickly obtain the tissue samples to minimize any effect on gene expression changes. All experiments were performed according to regulations and guidelines established by this committee.

### Animal Samples

All birds were maintained in the China Agricultural University poultry resources station, with free access to standard feed and water. Uterus, isthmus, hypothalamus, hypophysis, heart, liver, spleen, kidney, pectoral muscles, pancreas, magnum, ovary, jejunum, cerebrum and cerebellum were harvested from four normal White Leghorn hens at 49 weeks of age. Uterus samples were also collected at different physiological stages (four birds at 13 weeks, 16 weeks, 20 weeks, 27 weeks, respectively), from birds which were reared under the same environmental conditions. Birds at 13, 16 and 20 weeks of age are not in the reproduction cycle and the birds at the same weeks of age were slaughtered at the same time of day. Birds at 27 weeks of age were slaughtered at 2 h following ovulation. In order to measure correlations between OC-17 mRNA expression level and eggshell quality, we collected eggs from a Rhode Island White Layer line which exhibits divergent eggshell strength. At 40 weeks of age, 3 eggs from each hen were collected for measuring eggshell quality traits. Eggshell strength and eggshell thickness were measured within 12 h after collecting eggs. The average value of 3 eggs per hen for each eggshell quality trait was used in the following analysis. The eggshell strength and eggshell thickness were measured as published before [12]. Hens were rank ordered according to eggshell strength. We sampled the actively calcifying uterus (22 h after ovulation, egg with eggshell) of the top 8 hens (highest eggshell strength) and bottom 8 hens (lowest eggshell strength) for mRNA expression analysis. The entire uterus was taken out and rapidly wiped to remove adhering fluid. The middle part of the uterus was excised with scissors and transferred to a tube. The tube was immediately plunged into liquid nitrogen and then stored at −80°C until RNA extraction.

### RNA-Seq and Bioinformatics Analysis

Uterus tissue (active uterus, 22 h after ovulation, egg with eggshell) was obtained from one White Leghorn hen at 49 weeks, and used for RNA-Seq. Approximately 10 µg of sheared cDNA were prepared for Illumina sequencing according to the manufacturer's protocols. Paired-End libraries were prepared from a 200–230 bp size-selected fraction following adapter ligation and agarose gel separation. The library was sequenced using a multiplexed paired ends protocol with 101 bp of data collected per run on the Illumina Hiseq 2000 (the data are archived at the NIH Short Read Archive under accession number SRX180570 http://ncbi.nlm.nih.gov/sra). Base calling was performed by the Illumina instrument software.

The reads quality value (Q-value) distribution showed that 5–15 base quality in the 5′-end was lower than Q20. All reads were trimmed by 20 base pairs at the 5′-end to keep the same length using FASTX-Toolkit (http://hannonlab.cshl.edu/fastx_toolkit/). Trimmed reads were used in the following bioinformatics analysis. Trinity [13] software (version: r20131110) was used to conduct *de novo* transcriptome assembly with default parameters. We only kept contigs with length longer than 300 bp for further BLAST analysis [14]. The putative signal peptide was predicted using SignalP 4.0 [15].

**Figure 1:**
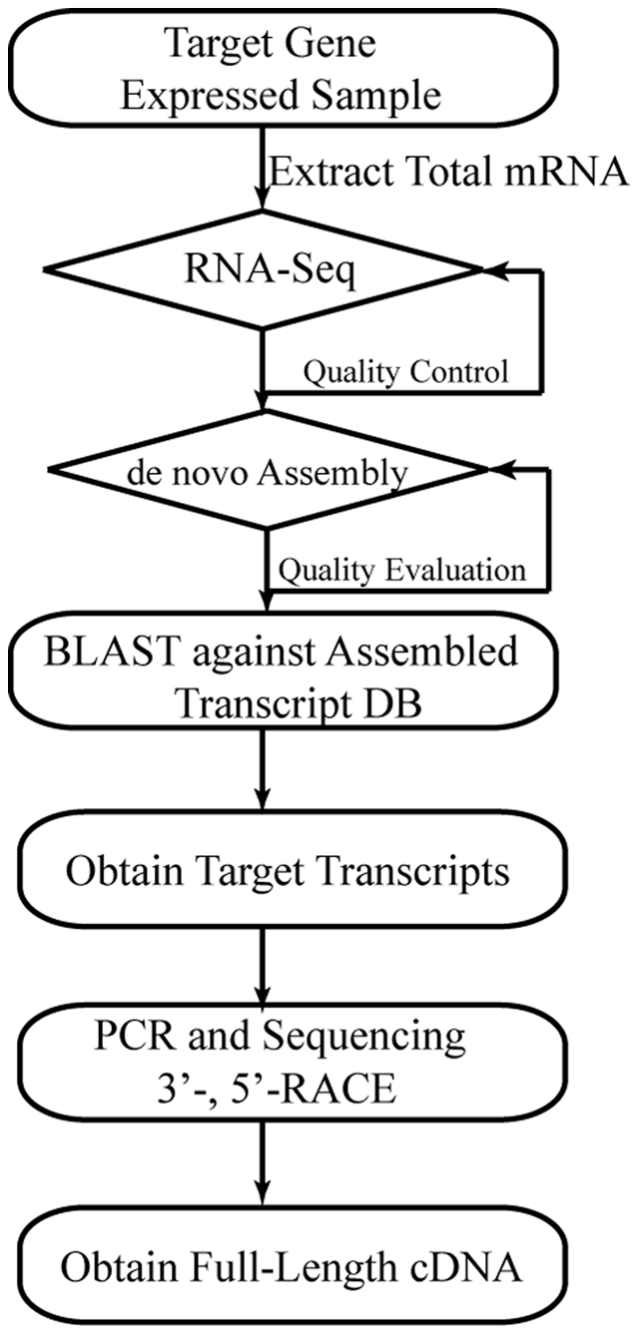
The *de novo* transcriptome assembly and cloning pipeline. This pipeline has four key steps: (1) Sampling the suitable tissues. Target genes must be relatively highly expressed in the sampled tissues; (2) Transcriptome assembling. The *de novo* assembly is necessary to obtain the target gene whether we have the reference genome or not; (3) Alignment of the partial target gene protein/EST to the assembled transcripts. We used the reference OC-17 protein sequence (GenBank No.: Q9PRS8) to conduct tBLASTn against the assembled transcripts. (4) RACE. The last step is to use RACE to confirm the assembled transcripts and obtain the full-length cDNA.

We used bowtie2 (version 2.2.0) [16] to map short-reads on the candidate OC-17 transcript with the default parameters. SAMTools (version 0.1.19) [17] was used to process the alignment file. SeqMonk (version: 0.27.0) package (http://www.bioinformatics.bbsrc.ac.uk/) was used to quantify and display the coverage of the OC-17 transcript from the short-reads alignment file. Only reads with mapping quality>5 were used in the coverage analysis. The pipeline to obtain full-length OC-17 cDNA is shown in Figure 1.

### Cloning Full-Length cDNA and Quantitative Expression Analysis

**Figure 2:**
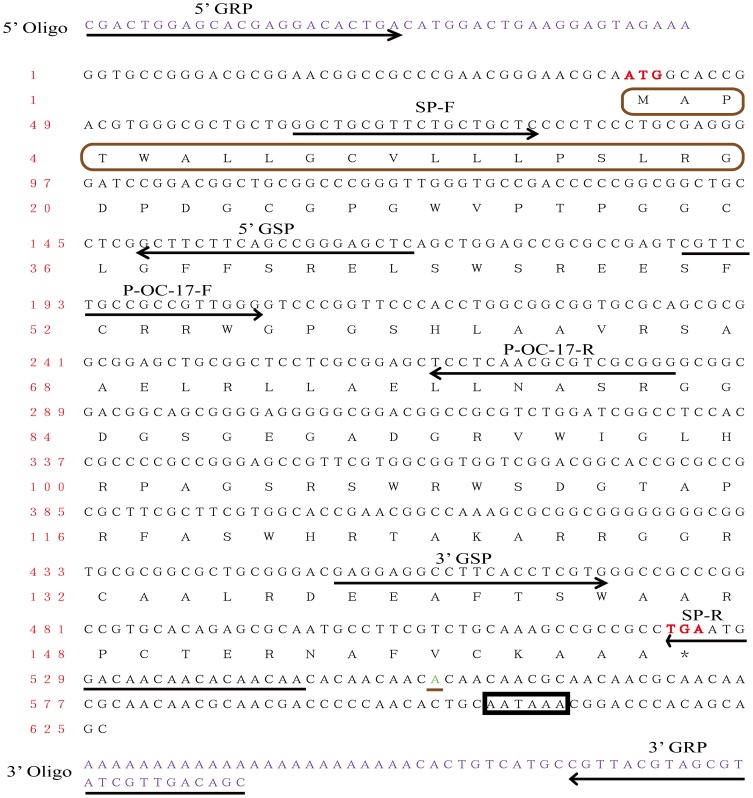
RACE strategies for cloning Ovocleidin-17 and nucleotide and deduced amino acid sequence of OC-17. The protocol of the SuperScriptTMIII RT Kit (Invitrogen Life Technologies Bio Inc.) was followed. Gene RACE Primers (GRP) anneal to the adaptor sequence, and Gene Special Primers (GSP) were designed to be complementary to the Ovocleidin-17 cDNA. The start and stop codons of the open reading frame are marked in red color. Black box indicates the poly-adenylation signal (AATAAA). The nucleotides marked with red color were obtained by RACE. Brown box represented the extra 19 deduced amino acids compared with published OC-17 amino acids (Q9PRS8), which is a putative signal peptide. Note that there is a one nucleotide difference (green color with black underline) between the RACE cDNA and the assembled transcript.

Total RNA was extracted from uterus with the E.Z.N.A™ Total RNA Kit (OMEGA Bio-tek Inc., USA) according to the manufacturer's protocol. The quality of extracted RNA was checked by agarose gel electrophoresis and the concentration was estimated by spectrophotometer. The RACE strategies are shown in Figure 2 and the corresponding primers are presented in Table 1.

**Table 1:** Primers for amplifying cDNA and quantifying mRNA of OC17.

Primer category	Primer name	Nucleotide sequences	Size (bp)
**RACE primers**	5′GRP	5′-CGACTGGAGCACGAGGACACTGA-3′	212
	5′GSP	5′-GAGCTCCCGGCTGAAGAAGC-3′	
	3′GRP	5′-GCTGTCAACGATACGCTACGTAACG-3′	235
	3′GSP	5′-GAGGAGGCCTTCACCTCGTG-3′	
	SP-F	5′-GGCTGCGTTCTGCTGCTC-3′	481
	SP-R	5′-TTGTTGTGTTGTTGTCCATTCA-3′	
	5′Oligo	5′-CGACTGGAGCACGAGGACACTGACATGGACTGAAGGAGTAGAAA-3′	44
	3′Oligo	5′-GCTGTCAACGATACGCTACGTAACGGCATGACAGTGTTTTTTTTTTTTTTTTTTTTTTTT-3′	60
**qRT-PCR primers**	P-*OC-17*-F	5′-CGTTCTGCCGCCGTTGGG-3′	96
	P-*OC-17*-R	5′-CCCGCGACGCGTTGAGGA-3′	
	P-*β-Actin* -F	5′-TATGTGCAAGGCCGGTTTC-3′	110
	P-*β-Actin* -R	5′-TGTCTTTCTGGCCCATACCAA-3′	

GRP means Gene RACE Primer which corresponded to the adaptor sequence and GSP means Gene Special Primer which was complementary to the OC17 cDNA. Primers for each sequence end are indicated either 5′ or 3′. Forward primers and reverse primers for qRT-PCR have prefixes ‘F’ and ‘R’, respectively. 5′GRP and 5′GSP were used for the cloning of 5′ end of the OC17 cDNA while 3′GRP and 3′GSP were used for the 3′ end. SP-F and SP-R were used for validation of the other parts of the OC17 cDNA. 5′Oligo and 3′Oligo were provided by the Gene Racer Kit.

The RACE experiment followed the protocol of the Gene Racer Kit (Invitrogen Life Technologies Bio Inc.). Gene RACE Primers (GRP) and Gene Special Primers (GSP), prepared to be complementary to the OC17 cDNA, were used as a primer set to amplify the cDNA sequence by PCR (Table 1). Primers for each sequence end were prefixed with 5′ or 3′ for the corresponding GRP and GSP (for instance, 5′GRP and 5′GSP) and. Nested Primers (N-GRP and N-GSP) were used in case the GRP and GSP primer set could not generate a satisfactory product. In addition, another primer set (SP) was designed to complete the entire cDNA sequence of OC17, as the products of the corresponding RACE primer sets could not complete the entire sequence. PCR products were cloned and sequenced.

Quantitative real-time PCR was employed to detect temporal and spatial expression of chicken OC-17. Chicken*β-Actin* (GenBank Accession ID: NM_204305) served as a housekeeping gene. Primer 3 Input (v. 0.4.0) was used with default parameters to generate primer pairs for selected transcripts (Table 1). Total RNA was extracted from different tissues as described before. The total RNA (50 ng/ul) was reverse transcribed using M-MLV reverse transcriptase (Promega Corporation), as recommended by the supplier. Quantitative real-time PCR was performed using the SYBR Green Master Mix (Life Technologies) on ABI 7500 Real Time System (Applied Biosystems, USA). The experiments were carried out in triplicate. The cycling conditions were 95°C for 5 min, followed by 40 cycles at 95°C for 15 s and 60°C for 1 min. This experiment used the 2^−ΔΔCT^ method to analyze the relative changes in gene expression from quantitative real-time PCR experiments.

We used the one way ANOVA analysis to analyze the means differences among samples. Then we applied pairwise t-test to compare OC-17 expression between two tissue/time points. All statistical analyses (avo function and pairwise.t.test functions in R) were conducted in the R computation environment (http://www.r-project.org), using p<0.05 as a cutoff.

## Results and Discussion

### Transcriptome Assembly: Successfully Assembling Target Transcripts

The tBLASTn results confirmed that the OC-17 gene is located in the gap region of the chicken reference genome. This means that we could not use mapping-based methods to align short-reads onto the reference genome. As OC-17 protein is predominantly present in the adult hen uterus during eggshell formation, we collected a mature hen uterus sample to process for RNA-Seq. This approach yielded 37,390,288 paired-end reads (GenBank SRA NO.: SRX180570). We used the Trinity package to conduct *de novo* assembly for uterus RNA-Seq data to obtain the transcripts. This resulted in 76,068 transcripts, ranging in length from 201–13,003 bp with 423 bp median length. The N50 of the assembled transcript is 1,172 bp. We only kept contigs with a length longer than 300 bp. The final assembled contig dataset which was used for BLAST analysis has 51,512 contigs with 649 bp median length.

We used the published OC-17 amino acid sequence (GenBank No: Q9PRS8) to perform tBLASTn against the assembled uterine expressed contigs. The analysis pipeline that we developed is shown in Figure 1. The tBLASTn results revealed that OC-17 amino acid sequences were fully covered by two contigs. The longer contig almost fully covered the shorter contig except for a 10-bases difference. We used the long contig as the assembled OC-17 transcript for the following analysis and primer design for molecular cloning. We also plotted the reads distribution on the assembled OC-17 transcript and found the reads were evenly distributed across the entire OC-17 transcript (Figure S1). In total, there 120,023 reads were mapped on the assembled OC-17 transcript which represented very high coverage. Alignments showed that the assembled transcripts not only included all the reference OC-17 amino acid sequence, but also included an extra deduced 19 amino acids at the N-terminus of the mature protein (Figure 2, brown box). Analysis of the new sequence with the SignalP 4.1 server [16] (http://www.cbs.dtu.dk/services/SignalP/) revealed that a signal peptide cleavage site is predicted between residues 19 and 20, suggesting that the extra amino acids correspond to a putative signal peptide commencing with the methionine start codon. This is consistent with OC-17's function as a secreted extracellular matrix protein [10]. The GC content of the candidate OC-17 transcript is 72.17%, which is likely one of the major reasons why researchers have not obtained the OC-17 mRNA sequence. Another major reason is that OC-17 only shares very limited conserved amino acid sequences with other related proteins in the GenBank database. Several homologous proteins in different avian species have been isolated from eggshell and sequenced: rheacalcin, rhea [18], dromaiocalcin, emu [18], ansocalcin, goose [19], struthiocalcin, ostrich [20] and ovocleidin-17, chicken [10]. Sequence analysis showed that these proteins all belong to a family of C-type lectin-like proteins [18]. However, OC-17 only showed 32–47% sequence identity with the other identified matrix proteins [18]. Furthermore, none of the cDNA sequences for these eggshell matrix proteins are yet available. It is difficult to design degenerate primers for cDNA cloning based on poor multiple protein sequence alignments. All of these reasons have hindered previous attempts to obtain the OC-17 mRNA sequence.

Development of the next-generation sequencing technology makes it easier than before to obtain tissue transcriptomes for model and non-model organisms. Transcriptome *de novo* assembly has been widely used in the analysis of non-model organisms [21], [22], but less so for model organisms. If we tried to map RNA-Seq short-reads to the chicken reference genome, we would never be successful in assembling transcripts located in the gapped regions. Therefore, we also need to do *de novo* transcriptome assembly for species for which a reference genome exists, for this purpose. There are a number of situations that require this approach for integrating transcriptome assembly and RACE to obtain target full-length mRNA. Firstly, targeted genes may be located in the gapped/mis-assembled reference genome region. Secondly, there may be species-specific target genes which do not have high similarity to orthologs in other species. Thirdly, target genes may correspond to purified and sequenced protein products that fail to be cloned using RACE based on the partial amino acid sequences. In this specific example, once we obtained the target gene cDNA, it was necessary to design primers based on our assembled OC-17 transcript sequence and apply RACE to confirm the accuracy of the transcriptome assembly.

### Full-Length cDNA confirmed the Accuracy of Transcriptome Assembly

Based on the assembled transcripts, gene-specific primers (Table 1, Figure 2) were designed to amplify the full-length cDNA of chicken OC-17 from chicken uterus. We used the 3′-RACE and 5′-RACE kit to clone the 3′- and 5′- sequences, respectively. The full-length OC-17 cDNA is 626 bp in length, with an open reading frame of 483 bp (GenBank Accession No.: KF835610; Figure 2). Sequence analyses confirmed that full-length OC-17 cDNA included the extra 19 amino acids corresponding to the putative signal peptide, compared to the previously published OC-17 amino acid sequence (Figure 2, with brown box). The RACE cDNA was the same as the assembled contigs, and showed the power of *de novo* assembly in discovering transcripts for species with/without a reference genome. The newly obtained OC-17 cDNA sequence can now be exploited for mRNA-related studies. OC-17 may be critically important in CaCO_3_ transformation and crystal growth [9], [11], and also in the regulation of eggshell quality [23]. However, the amino acids that directly interact with the mineral are not yet known. The OC-17 cDNA sequence can now be used to introduce mutated sites to produce recombinant proteins to verify the functional consequences for CaCO_3_ crystal growth *in vitro*.

We explored the relationship between eggshell mineralization and OC-17 by investigating OC-17 expression at different hen uterus developmental stages, and determining the specificity of OC-17 expression.

### Expression Analyses of OC-17 Supports its association with Biomineralization

Although thickness is the main factor contributing to mechanical strength of the eggshell, the structural organization of the eggshell at different levels has a significant effect. Ultrastructure (the organization of major structural units) and texture (the size of crystals, their shape and crystallographic orientation) are especially important. The soluble matrix proteins of calcitic biomaterials can modify crystal growth, and thus regulate the macroscopic properties of the resulting bioceramic [8], [24].

**Figure 3:**
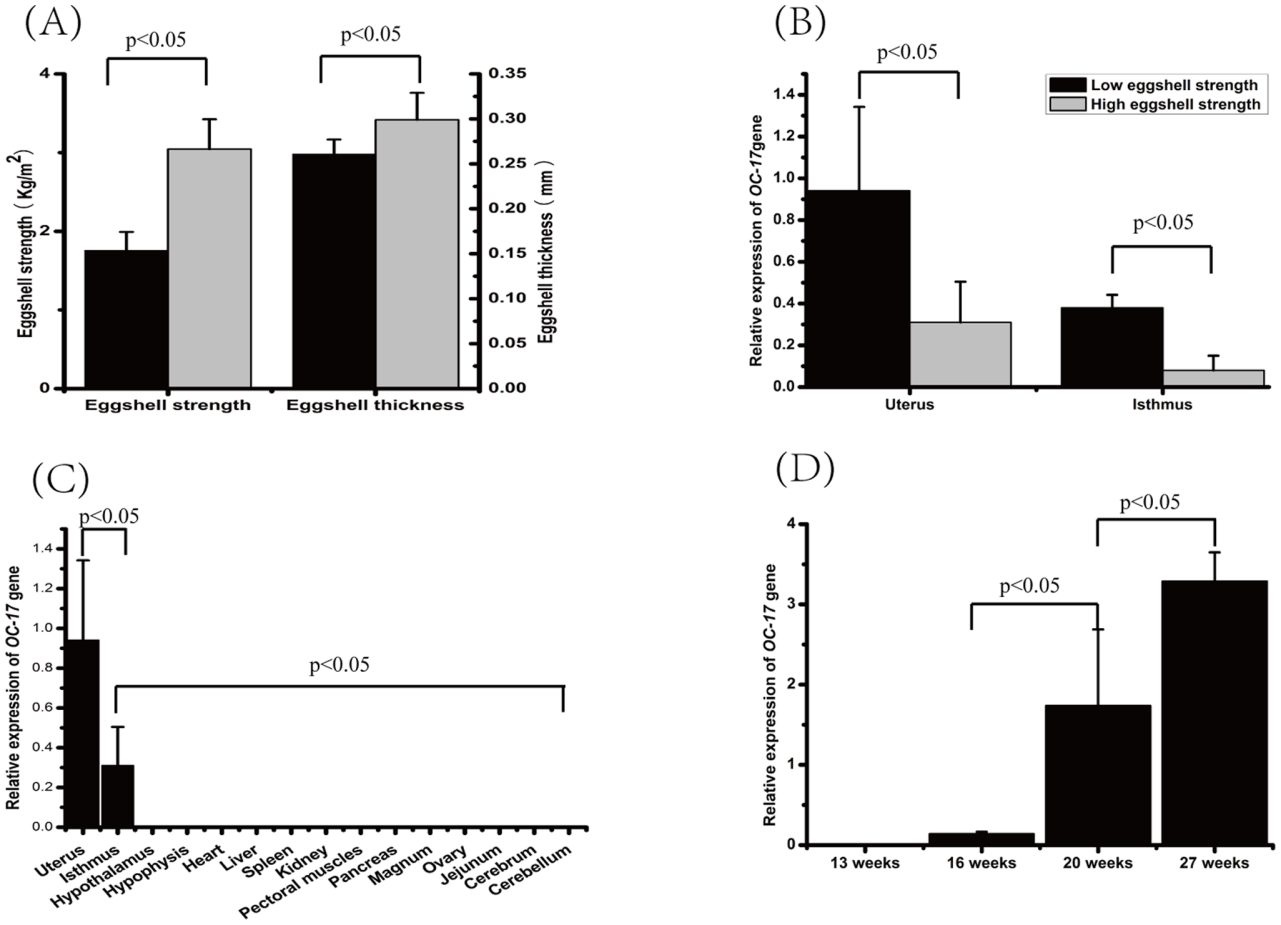
Eggshell biomechanical measurements and expression analysis of Ovocleidin-17. (A) Eggshell phenotypes showed significant differences between the two tails of the eggshell strength groups; (B) the OC-17 mRNA expression levels were significantly higher in hen uterus and isthmus in the low eggshell strength groups compared with the high eggshell strength groups; (C) OC-17 mRNA can only be detected in hen uterus and isthmus among the 15 sampled adult hen tissues. One way ANOVA analysis suggested there were significant expression differences among different tissues. OC-17 showed a much higher expression level in uterus than in other tissues (pairwise t-test, P value<0.05); (D) One way ANOVA analysis suggested that significant expression differences existed among different stages. OC-17 showed an increasing pattern of expression between the immature and mature laying stages (pairwise t-test, P value<0.05).

We quantified uterus and isthmus OC-17 expression level in two hen groups laying eggs with different eggshell strength (Figure 3A). The OC-17 mRNA expression level in the high eggshell strength group is significantly lower than in the low eggshell strength group (Figure 3B). This is consistent with the OC-17 protein concentrations in eggshell of eggs laid by young and old hens, two groups where eggshell strength is significantly different [23]. This suggested that OC-17 protein concentration might be negatively correlated with eggshell strength, as originally reported [25].

As tissue-specific gene expression can indicate gene function in certain tissues [26], we also wanted to determine the OC-17 mRNA expression status in a variety of tissues. Among all the 15 investigated tissues, OC-17 was only detected in uterus and isthmus (Figure 3C). OC-17 has been considered as a uterine-specific protein based on Western blotting [10], although sensitive proteomic studies have detected low levels of this protein in other egg compartments, such as egg white [27], [28], vitelline membrane [29] and egg yolk plasma and granules [30]. Indeed, we also detected expression of the OC-17 in the isthmus, the oviduct segment where the eggshell membranes are synthesized. Does the OC-17 expression correlate with uterine developmental stage? To address this question, uterine tissue from four white leghorn hens at each stage (developing, immature uterus: 13 weeks, 16 weeks and 20 weeks; actively laying uterus: 27-weeks) were harvested and analyzed by qRT-PCR. The OC-17 gene was highly expressed in mature uterus of 27-week hens and much less expressed in the immature uterus of 13-week, 16-week and 20-week hens (Figure 3D). OC-17 showed an increasing trend of expression during the progressive developmental and laying stages.

OC-17 begins to be expressed during the uterine developmental stages (13-week, 16-week), albeit at low levels. Does this mean that OC-17 has functions during development of the immature uterus? Further studies are needed to explore OC-17 potential functions in hen uterine development. In summary, this study provides new evidence that OC-17 is expressed in a hen oviduct-specific fashion, and may have potential new roles during uterine development. Moreover, its expression levels in the mature, laying uterus are inversely correlated with eggshell quality.

## Conclusions

We have demonstrated that *de novo* transcriptome assembly can be critically important for certain purposes in model organisms. This study is the first one to report the full-length OC-17 cDNA sequence, and provides preliminary data describing physiological regulation of its expression, as a foundation for OC-17 mRNA related studies. Furthermore, we provide a general method for biologists experiencing difficulty in obtaining candidate gene full-length cDNA sequences.

## Supporting Information

Figure S1 Coverage plot of the OC-17 transcript. **Red color represents the reads from the 5′-end while the blue color represents reads from the 3′-end. Last row represents the coverage plot of the mapped short-reads on the OC-17 transcript.**
(TIF)
