# Supplementary figures and images for: Integrating De Novo Transcriptome Assembly and Cloning to Obtain Chicken Ovocleidin-17 Full-Length cDNA
Source: PLoS One. 2014 Mar 27;9(3):e93452. doi: 10.1371/journal.pone.0093452 (PMC3968166; doi:10.1371/journal.pone.0093452)

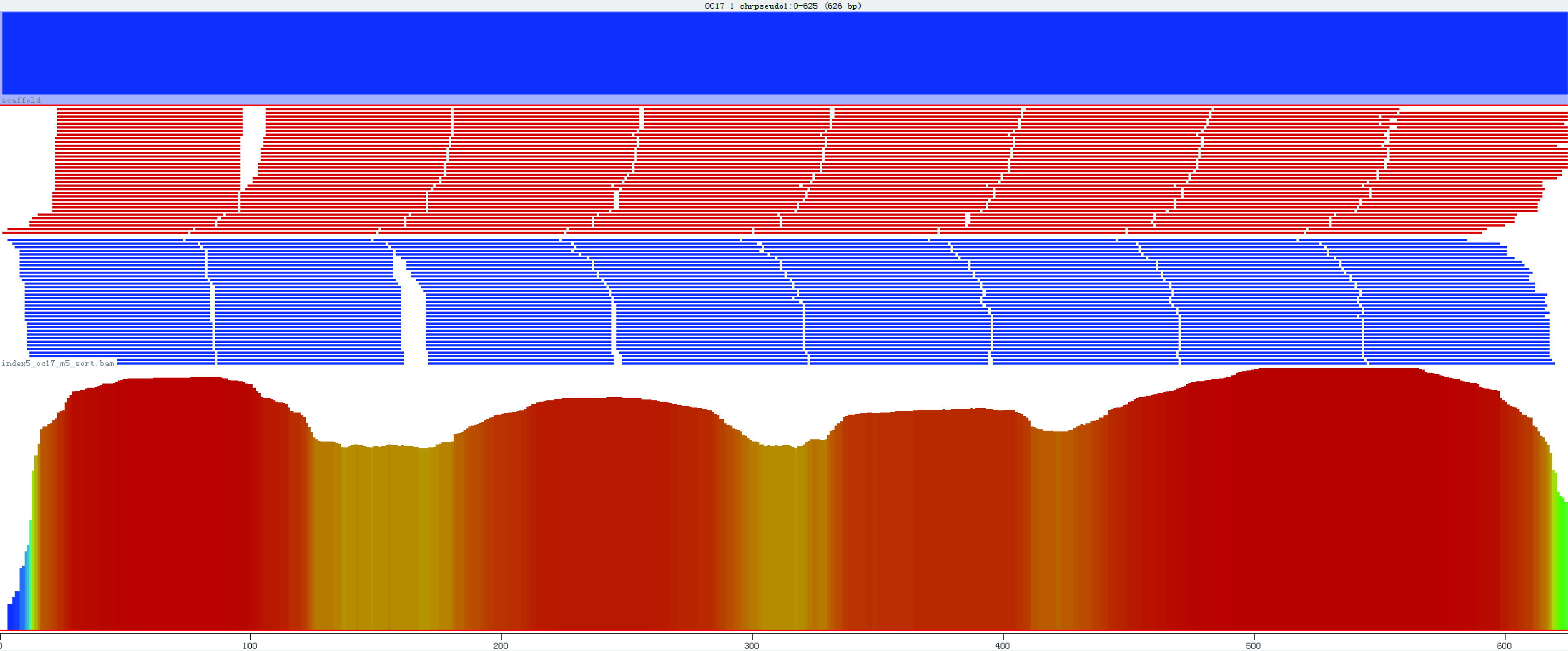

Supplement: Figure S1 — Coverage plot of the OC-17 transcript. Red color represents the reads from the 5′-end while the blue color represents reads from the 3′-end. Last row represents the coverage plot of the mapped short-reads on the OC-17 transcript. (TIF) [file pone.0093452.s001.tif]
